# Exploiting Plant–Natural Enemy Interactions: Selection and Evaluation of Plants for the Improvement of Biological Control

**DOI:** 10.3390/insects16020138

**Published:** 2025-01-31

**Authors:** Hipolito Cortez-Madrigal

**Affiliations:** Instituto Politécnico Nacional-CIIDIR, Unidad Michoacán, Jiquilpan de Juárez 59510, Mexico; hcortezm@ipn.mx

**Keywords:** parasitoid, conservation, alternate food, nectar, pollen

## Abstract

To identify and select wild plant species as a source of nectar and pollen for the conservation of natural enemies (NEs), with an emphasis on parasitic Hymenoptera, entomological samplings were carried out on the inflorescences of plants in the northwest of Michoacán, Mexico, in 2020. The species were selected based on the number and diversity of NEs, flowering period, phytosanitary risks (pests), and ease of multiplication. Of more than 27 plant species, NEs were recorded in 24. The Eulophid family represented 74.14% of 23 families of parasitoids recorded, and in 19 of the 23 plant species with parasitoids, the eulophid family was the most prevalent. Thirteen plant species were selected; annual species such as *Tithonia tubaeformis* and *Stevia serrata* stand out. Among the shrubs, *Senecio salignus* and *Baccharis salicifolia* stand out, as do trees such as *Viguiera quinqueradiata*, *Thouinia villosa* and *Buddleja parviflora*. *Serjania racemosa* and *Phytolacca icosandra* stand out for their long flowering period (>5 months) and the wide diversity of beneficial entomofauna. They are plants that reproduce easily and have a low phytosanitary risk to crops. The presence of flowering plants in agroecosystems could attract and maintain NE populations, even before pests arrive, with predictable benefits for integrated pest management.

## Simple Summary

Beneficial fauna in agriculture is facing problems in today’s farming system, mainly due to monoculture and the irrational use of agrochemicals. A sustainable solution must be based on exploiting the ecological interactions that occur in ecosystems. For instance, different natural enemy (NEs) species need nectar and pollen as alternative foods to complete their life cycle. Therefore, the exploration and selection of native nectariferous plants must be an important consideration for better biological pest control using NEs. For one year, plant species native to Northwest Michoacán, Mexico, that hosted the highest numbers of NEs were explored. Twenty-three families of parasitoids and other predators were recorded, with few records of phytophagous insects (one to four). Thirteen species of flowering plants with the highest potential for the conservation of NEs were selected. The plants used are easy to reproduce and have complementary flowering periods that can maintain NEs populations throughout the year. The results could be helpful to enhance biological pest control and reduce the use of insecticides in agroecosystems.

## 1. Introduction

There is a consensus that the current agricultural production paradigm derived from the “green revolution” conflicts with beneficial insect populations, mainly due to the use and abuse of agrochemicals in the form of insecticides, fungicides, and herbicides [1,2]. In addition to its direct effect, the use of residual herbicides for the persistent suppression of weeds indirectly disturbs the populations of beneficial entomofauna, such as natural enemies (NEs) and pollinators [2,3]. For example, in Mexico, it is estimated that around 85% of cultivated and wild plants require pollination by different organisms. However, a pollination crisis is also recognized in different regions of the world, presumably due to the high degree of anthropization of natural landscapes [4]. Furthermore, large extensions of monocultures promote an imbalance between pest and NEs populations, where the former, in the absence of the latter, increase their populations to levels of economic damage [5].

Contradictorily, agriculture, as a human activity, is highly dependent on entomophilous pollination and regulators of phytophagous insects (i.e., pests), but also, it is one of the environments that contribute the most to the reduction in their populations [1,2,6,7]. In response, various strategies have been implemented; among them are the deliberate use of cultivated pollinators, e.g., bumblebees and *Apis mellifera* L. [8], and the mass production and subsequent release of NEs for biological control [9]. However, the above are palliative measures since they only impact the effects, but not the causes of the pest problem.

A sustainable solution should be based on increasing ecological niches through plant diversification in agroecosystems [2,10,11]. Several studies show that plant diversification, whether within or around agroecosystems, can restore pollinator and NE populations and the benefits derived from them [12,13,14], as the Food and Agriculture Organization of the United Nations (FAO) [1] suggested.

In Mexico, there are isolated studies on pollinators and their conservation [15], but regarding the conservation of NEs in agroecosystems, it is evident that much remains to be done [16]. What can be observed in the field is that Mexican agriculture still maintains (for various reasons) wild or semi-domesticated flora close to crops; however, its importance in the conservation of beneficial insects has not been well documented. If the expansion of monoculture and the irrational use of herbicides continue, part of this wild vegetation will likely be lost before its importance is assessed.

According to the above, one could consider selecting wild plant species with agro-beekeeping potential, that is, those beneficial for beekeeping production, but also that provide other services to agriculture, such as source of nectar, pollen [2,17] and alternative hosts [18,19] for increase and conservation of NEs.

Since 2015, in the northwest region of the state of Michoacán, México, studies have been carried out to identify, select, and exploit wild plant species that may be useful in the conservation of beneficial insects, including species of nectariferous plants. It is assumed that all species of flowering plants can be equally useful for the conservation of pollinators and NEs [2,20], and this assumption is partially true. For example, out of 90 flowering species in the northwest of Michoacán, only 27 (30%) recorded *Apis mellifera* L., with varying visitation levels, from 0.2 to 1.68/plant species [21]. If *A. mellifera* is considered a guide to the identification of nectariferous species, it is clear that not all flowering plant species are equally attractive to pollinators, which could also be true for entomophagous insects that similarly require nectar and pollen.

On the other hand, nectariferous plants should also be free of insects and pathogens that are potentially risky for crops. Of the 27 species of nectariferous plants selected using *A. mellifera*, there were few records of phytophagous insects; on the contrary, records of pollinators and NEs were common. Species with a higher rate of bee visits and floral temporality, such as *Serjania racemosa* (autumn–winter, 5 months) and *Phytolacca icosandra* (spring–summer–autumn–winter, 10 months), and with a low phytosanitary risk could be deliberately incorporated into agro-beekeeping production systems [21]. Their presence and conservation would provide important services to beekeeping production and pest regulation.

To achieve this, it is important to carry on research that provides more information on the potential of nectariferous plants in the conservation and increase in NEs that can regulate key crop pests. Based on this, the objective of the study was to evaluate and select native nectariferous plants from the northwest of Michoacán, Mexico, that possess the greatest potential for use as conservation banks and to increase the NEs of pests throughout different seasons of the year.

## 2. Materials and Methods

### 2.1. Study Region and Plant Species

**Figure 1:**
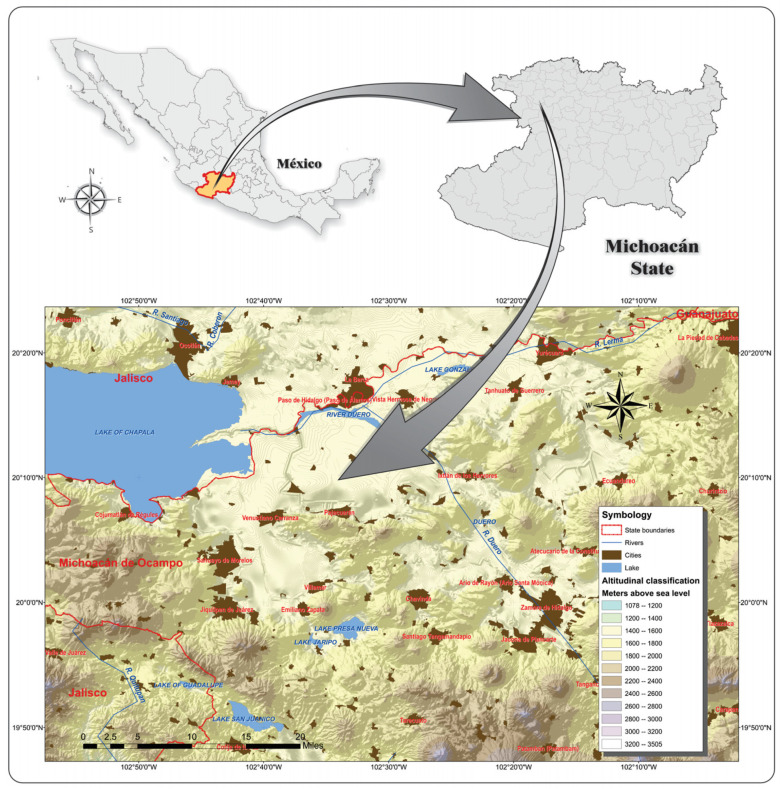
Location of the study region in the northwest of the Mexican state of Michoacán.

The work was carried out in the northwest of Michoacán, with emphasis on the Lerma–Chapala region and part of the Sierras and Bajíos Michoacanos province (Figure 1). Sampling sites included disturbed and natural habitats, such as vegetation bordering crops, plants on the edges of dirt roads and highways, agricultural areas, grasslands, cattle pastures and hills with native vegetation, and experimental land of CIIDIR-IPN, with altitudes from 1500 to 2000 masl.

The study started from a database of wild flowering plants (28 spp.) from the northwest of Michoacán, previously identified for its high nectariferous potential [21]. During the study, other species not previously documented were incorporated. Trees, annual plants, shrubs, and climbing plants were included; preference was given to native species. Comparatively, two of the most widely used plant species worldwide for the conservation and increase in NEs were included: sweet alyssum, *Lobularia maritima*; and cilantro, *Coriandrum sativum* [13].

### 2.2. Insect Sampling

Based on the selected plants, during 2020 and part of 2021, samplings were established in transects where the existence of nectariferous plants was known. The samplings began at 9 a.m. and ended at 1 p.m., and their frequency depended on the main flowering periods previously recorded [21]; once the plants were detected, they were visually examined for phytophagous insects, which were recorded qualitatively (absence or presence and damage); then, groups of inflorescences were vigorously shaken inside zipper-type plastic bags to collect the insects associated with the flowers. The sampling unit was 10 inflorescences, and the number of samples varied from two to nine, according to the distance, accessibility, plant density, and flowering season. Thus, for *Budleja*, only one sampling was carried out because it was in an area of difficult access on the top of a hill and with only four specimens. Records were expressed as the mean number of insects/10 inflorescences.

The material was transported to the laboratory, where the groups were reviewed and counted under a stereoscopic microscope (Carl Zeiss^®^, Oberkochen, BaWü, Germany), and then they were separated as phytophagous species and NEs [22], with a greater emphasis placed on the second group. Natural enemies were placed in 75% alcohol for later identification.

### 2.3. Plant Multiplication and Management

Seeds from plants with the greatest potential for attracting NEs were collected from the plant species, and their germination and development were evaluated. This phase was carried out in a greenhouse, where the seeds were individually sown in polystyrene trays with germinating peat (peat moss^®^, Colombier, QC, Canada); and, based in 100 seeds, the germination percentage was evaluated. The seedlings obtained were transplanted into plastic pots with a mixture of soil + peat moss, where they were preserved until their final transplant to the experimental field located in Jiquilpan, Michoacán (19°59′58.0″ N, 102°42′23.9″ W), at an altitude of 1550 masl. The climate is semi-warm and humid, with summer rains and a rainfall of 807 mm per year, and with average daily temperatures of 11.7 °C and 26.9 °C; the soil is vertisol type [23].

In some species, vegetative propagation was evaluated. Stem sections were placed together with a rooting solution (Rootex^®^, Cosmocel, Guadalajara, Jal., Mexico); some cuttings were placed in water, and others directly into soil. Once the emergence of roots was detected, the cuttings were transplanted into pots with soil before their final transplant to the experimental terrain.

The plants established were weekly checked for phytophagous insects. In addition, their type of growth was considered: tree, shrub, climber, or annual. Moreover, the flowering period was considered, so that the plants selected would guarantee flowering during most of the year in the agroecosystems.

### 2.4. Analysis of Results

Several of the plant species were identified in previous studies [21], while the new species were identified through the specialized literature [24] and Internet databases. In some cases, support was provided by Dr. Rubén Torres García (IPN-CIIDIR, Jiquilpan, Michoacán, Mexico). Insects were identified at the family level by means of the specialized literature [22,25,26] and by comparison with Internet databases.

To select the best plant species, a multi-criteria analysis was used [14]. The value resulting from the sum of the following variables was given: mean number of parasitoids, mean number of predators, number of parasitoid families, flowering period (in months); phytosanitary risk, where 0 = no insects recorded, and (−1), (−2)...(−n_i_) means one or more insect species and recorded damage; with status as weed being (−1); and multiplication of plant, where 0 = multiplication not achieved, and 1 = multiplication achieved. With these selection criteria, parasitoids had the greatest importance, followed by flowering period and phytosanitary risk, in that order.

## 3. Results

### 3.1. Plants with Records of Natural Enemies

In total, 26 species were identified within 10 plant families with records of NEs associated with their inflorescences: 11 were annual and 15 perennials. Three exotic species were included: *Rapistrum rugosum*, *Lobularia maritima*, and *Coriandrum sativum*; as well as *Tithonia diversifolia,* native to Southern Mexico. The family with the most records was Asteraceae (14 spp.), followed by Fabaceae (3 spp.), Sapindaceae (2 spp.), and Scrophulariaceae (2 spp.) (Table 1).

### 3.2. Entomophagous Associated with Inflorescences

Predators. Thirteen families of the orders Neuroptera, Hemiptera, Coleoptera, Diptera, and Hymenoptera were recorded. The Anthocoridae and Syrphidae families stood out. Other groups recorded in smaller numbers were members of the Coccinellidae, Chrysopidae, Staphylinidae, and Phymatidae families (Table 2).

Parasitoids. Twenty-three families of parasitic Hymenoptera of interest in biological control were recorded, either because they parasitize insects or because they are important plant regulators (e.g., weeds). The families of parasitoids were placed within eight superfamilies: Chalcidoidea (13), Platygastroidea (2), Ichneumonoidea (2), Chrysidoidea (1), Cynipoidea (2), Proctotrupoidea (1), Ceraphronoidea (1), and Apoidea (1) (Table 3).

More than 74% of the collected specimens belonged to the Eulophidae family, followed by Platygastridae (8.42%). Other important parasitic families were Pteromalidae, Encyrtidae, and Scelionidae (2.16% each); and Mymaridae and Bethylidae (1.8% each). Other families showed low and sporadic records (1–4 individuals; Table 3); sometimes they were recorded only in one sample. There were low records of the Ichneumonidae and Braconidae families. The Chalcididae, Leucospidae, and Signiphoridae parasitoid families were absent.

### 3.3. Plant–Natural Enemy Relationship

**Figure 2:**
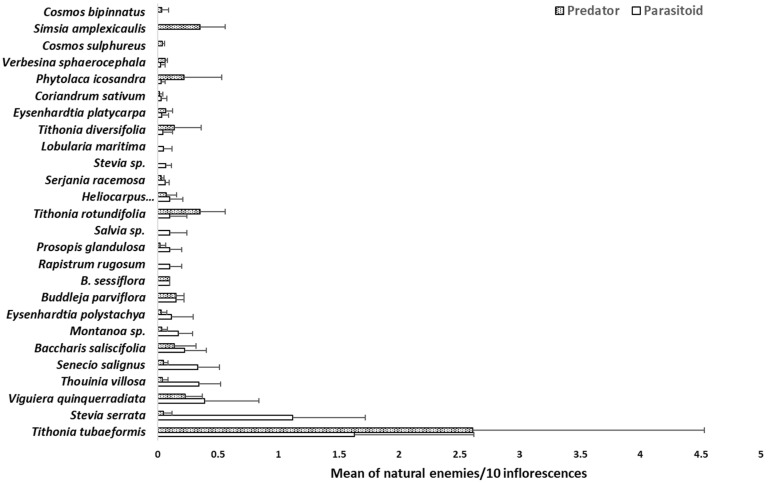
Mean number of natural enemies recorded on flowering plants in Northwest Michoacán, Mexico, 2020. Lines above the bar are the standard deviation.

The annual species *Tithonia tubaeformis* recorded the highest mean numbers of NEs/10 inflorescences, both parasitoids and predators; then, *S. serrata* stood out, followed by two tree species, *V. quinqueradiata* and *T. villosa*. In contrast, the species *Cosmos* spp. and *V. sphaerocephala* showed the lowest records; however, compared to the exotic species, *L. maritima* and *C. sativum*, the attraction of NEs to native species was similar and, in several cases, superior to the exotic species in the attraction of NEs (Figure 2).

*Cosmos bipinnatus, C. sulphureus*, and *S. amplexicaulis* only recorded predators; and in some cases, such as *T. tubaeformis*, the predator record was abundant, particularly from the Anthocoridae family. In contrast, species such as *R. rugosum, Salvia* sp., *Stevia* sp., and *L. maritima* only recorded parasitic species (Figure 2).

**Figure 3:**
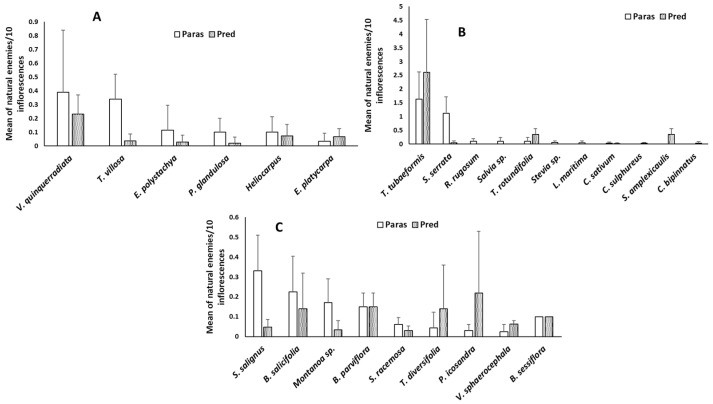
Mean number of natural enemies by plant groups in the northwest of Michoacán, Mex. (**A**) Trees, (**B**) annuals, and (**C**) shrubs and climbers. Lines over bars is standard deviation. Pred = predator; paras = parasitoid.

A graphic analysis by growth type (trees, shrubs, climbers, and annuals) shows that all seven-tree species evaluated played an important role in attracting NEs, where *V. quinqueradiata* showed a tendency towards a higher average of insects. *T. villosa* ranked second among nectariferous tree species, followed by *E. polystachya* and *P. glandulosa* (Figure 3A).

Regarding annual plants, according to the average number of insects, the most attractive was *T. tubaeformis*. The species with no records of parasitoids were *S. amplexicaulis* and *Cosmos* spp. In contrast, predators were absent in samples from *L. maritima*, *R. rugosum*, and *Salvia* sp. (Figure 3B).

**Figure 4:**
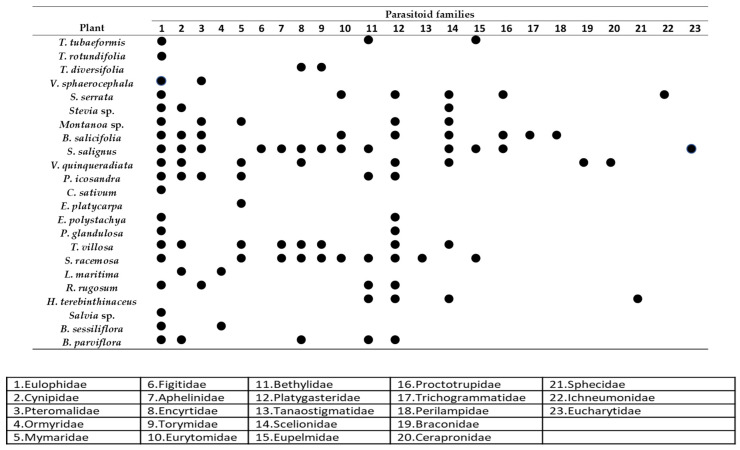
Distribution of families of parasitic Hymenoptera in different species of nectariferous plants in the northwest of Michoacán, Mexico.

Among shrubs and climbers, the species *S. salignus* and *B. salicifolia* recorded the highest average number of NEs. Although with modest records in terms of the average number of NEs (Figure 3C), *S. racemosa* ranked second among shrubs and climbers in terms of parasitoid diversity, with 10 families (Figure 4). Additionally, it is a species with no records of damage from phytophagous insects, so it represents a low phytosanitary risk to crops.

Although in previous studies, it was one of the most attractive species for *A. mellifera*, based on the mean of NEs, *V. sphaerocephala* was one of the least attractive plants (Figure 3C).

### 3.4. Parasitoid Diversity

Out of 23 families of microhymenoptera, Eulophidae was the most prevalent, with 74.1% of the total of parasitoids (Table 3). The plant species with the highest diversity of parasitic Hymenoptera were *S. salignus*, with thirteen families; *S. racemosa*, with ten; *B. salicifolia*, with nine; *V. quinqueradiata*, with eight; *T. villosa*, with seven; *P. icosandra* and *S. serrata*, with six; and *B. parviflora* and *Montanoa* sp., with five families each. The other species ranged from 1 to 3 families (Figure 4).

Eulophidae was the family with the highest presence in plant species, with records in 19 (82.6%) of the 23 plant species that recorded parasitoids, followed by Platygastridae, with 12 plants (52.17%). Other families with significant records were Cynipidae and Scelionidae, with eight plants each; and Pteromalidae, Mymaridae, and Encyrtidae were collected in six species of plants. The families that were only recorded in one plant were Figitidae, Eucharitidae, Ichneumonidae, Sphecidae, Ceraphronidae, Perilampidae, Braconidae, Tanaostigmatidae, and Trichogrammatidae (Figure 4).

### 3.5. Phytophagous Insects

There were few records of phytophagous insects in which damage to the sampled plants was confirmed. *Verbesina* was the species with the greatest diversity of phytophagous insects; among them were the aphid *Macrosiphum* sp., spittlebugs (Cercopidae), mealybugs (Pseudococcidae), treehopper (Membracidae), leaf miners (Agromyzidae), and gall insects. In *Heliocarpus*, abundant populations of larvae of the genus *Arsenura* (Lepidoptera: Saturniidae) were recorded; sometimes, the trees were completely defoliated. Although there is no evidence that this insect feeds on other plants, the damage that the insect causes to the trees is significant, which could reduce flower production.

In *B. salicifolia*, *S. salignus*, and *B. parviflora*, abundant colonies of aphids of the species *Aphis spiraecola* and *A. craccivora* were recorded. The damage caused was minimal, and the plants quickly recovered. In turn, the aphid colonies attracted and maintained a wide diversity of NEs; among them, Coccinellids, Chrysopidae, Syrphidae, and the Chamaemidae family were present among others. In *B. salicifolia*, the species *Membracis mexicana* (Hemiptera: Membracidae) was recorded. In *V. quinqueradiata,* the beetle, *Leptinotarsa behrensi* ssp. *puncticollis* (Coleoptera: Chrysomelidae), and specimens of the superfamily Psylloidea were present.

### 3.6. Plant Multiplication

Of the species evaluated, except for *T. villosa*, all were able to reproduce via seed. The percentage of germination varied from 8.3% in *T. tubaeformis* to 100% in the wild mustard, *R. rugosum*. *B. salicifolia* was easily multiplied by cutting, with 100% rooting. *Bursera fagaroides* and *B. bipinnata* (not evaluated in their flowering period) showed a differentiated take of cuttings: the first one was 100% in soil; in the second, only 20% of cuttings rooted (Table 4).

### 3.7. Selection of the Best Species

Based on the number and diversity of NEs, flowering period, phytosanitary risk (pests), and ease of multiplication, the best plants, from highest to lowest potential, were *P. icosandra*, *S. racemosa*, *S. salignus*, *B. salicifolia*, *Tithonia tubaeiformis*, *R. rugosum*, *V. quinqueradiata*, *P. parviflora*, *S. serrata*, *T. villosa, Montanoa* sp., *E. polystachya*, and *P. glandulosa* (Table 5). Except for *R. rugosum*, all are native plants; some grow abundantly in the region (e.g., *S. salignus*), while others must be multiplied to be incorporated into crops.

Another plant that attracted a diversity of parasitoid (10 families) was *S. racemosa*. Its abundance, long flowering period, and low phytosanitary risk placed it in second place. *Tithonia tubaeiformis* was the plant with the highest number of parasitoids and predators (Figure 2), but not the one with the greatest diversity (three families; Table 5).

## 4. Discussion

If, with climate change and its negative impact on food production, biological pest control becomes of fundamental importance, the conservation strategies of NEs must play multiple roles, for example, in the conservation of biodiversity, and derived from this, in the self-regulation of populations in its broadest conception [10,13]. Thus, the insertion and conservation of native nectariferous plants in agroecosystems on a rational basis is one of the main strategies for the conservation of NEs and pollinators [1,2,27].

Although there is worldwide interest in the conservation of NEs and pollinators [13,14,28,29,30], only a few plant species have been used, while native species have been relegated [13]. It is evident that more effort is needed when it comes to developing research, but, above all, even more effort is needed in its application for the conservation of NEs [11].

To our knowledge, in Mexico, this is the first effort to survey the native nectariferous flora for conservation-based biological control. In other regions of the world, nectariferous plants have been selected for biological control, but without mentioning the associated NEs [14,31]. However, not all flowering plant species are equally preferred, as demonstrated in the present study. For example, *C. bipinnatus* was one of the least preferred species, despite being recommended as a nectariferous species [29]. Likewise, not all flowering plants are equally attractive to all groups of NEs. Thus, plant species that recorded parasitoids were documented, but not predators and vice versa; likewise, some had the highest number, but not the greatest diversity; for example, *Tithonia tubaeformis* had the highest number, but only three parasitic families were recorded. In contrast, *S. salignus* and *S. racemosa* recorded the highest diversity, with 13 and 10 families, respectively, but not the highest number. This suggests that the potential of a plant in the conservation of NEs should be assessed both by the group and by the number and diversity of NEs it can attract and maintain.

*Serjania racemosa* is a climbing plant with vigorous growth. It is abundant in the region, has copious flowering for long periods (5 months), and was attractive to a wide variety of insects; in addition to NEs, pollinators, butterflies, bees, and various species of wasps were recorded. Farmers tolerate it on the edges of fields, where it grows on stone on fences.

Although *P. icosandra* maintained modest NEs records, its flowering during most of the year, including the dry season, makes it a species considered of great importance in NEs conservation. Additionally, it was a species with no records or damage from phytophagous insects, suggesting a low phytosanitary risk to crops. *V. sphaerocephala*, previously described as one of the most visited by *A. mellifera*, was one of the least attractive for NEs in the present study.

*Thouinia villosa* is a previously unnoticed tree as a nectariferous plant. This species occupied the second place among nectariferous tree species, while the sweetwood, *E. polystachya,* previously described as an important nectariferous species [21], in the present study, ranked third among trees in the attraction of NEs.

Interestingly, the iconic species of conservation biological control, *L. maritima* and *C. sativum* [13], were widely outperformed by most native plants in attracting predators and parasitoids. This means that there is great potential for the use of native nectariferous plants in NEs conservation. Unlike *L. maritima* and *C. sativum*, wild native plants are better adapted to adverse biotic and abiotic factors. Therefore, presumably less attention and investment are required for wild native plants than for cultivated ones, whether exotic or native.

The Anthocoridae family, commonly known as “pirate bugs” or “flower bugs”, stood out as predators in the inflorescences. The record of nymphs and adults demonstrates the importance of nectar and pollen for the survival of these predators in the absence of insect prey. Anthocoridae bugs are important natural regulators of phytophagous mites, such as the red spider *Tetranychus* spp.; thrips (Thysanoptera); whitefly (Hem: Aleyrodidae); and eggs and small larvae of lepidopterans and psyllids, among others [32]. *Tithonia* was an important reservoir of flower bugs.

Parasitoid diversity accounted for 63.8% (23 families) of the NEs. The recorded families include parasitoids of insects and spiders, and others are gall-formers in various plant species [33]. Therefore, from an ecological point of view, all recorded parasitic Hymenoptera insects can be considered biotic regulators of other organisms, including pests and weeds [26].

The diverse families of parasitoids documented here complement each other in population regulation, e.g., species of Trichogrammatidae, Mymaridae, and Scelionidae attack eggs; others attack nymphs, larvae, pupae, and adults [26]. The documented diversity of parasitoids should be greater if species are considered, since in the present study, they were identified only at the family level. Even so, the results obtained approximate the benefits that could be provided by the deliberate and rational conservation of nectariferous plants in agroecosystems.

The fact that some families of parasitoids have been recorded in low numbers and frequency does not mean that they are of little importance in pest control, nor that the plants have not attracted enough of them. What occurs is that some parasitoids are rare groups by nature, as surely are their hosts; for example, the Tanaostigmatidae family [22] was collected, in this case, on a single occasion. Also striking are the low records of the Ichneumonidae and Braconidae families, families of enormous importance in biological pest control [26].

Highly mobile insect groups are likely to have been poorly recorded by the sampling technique used, as was the case with Braconidae and Ichneumonidae; however, other techniques, such as visitation records, may underestimate the presence of less mobile species, such as most parasitic Microhymenoptera [34]. Given the diversity of parasitoid families recorded in this study, it could be considered that the sampling technique met the expectations of the study.

A requirement for the selection of companion plants is that they do not host crop pests [35]. However, it is difficult to find plant species without polyphagous species, e.g., aphids. The plants evaluated under field conditions had low records of phytophagous; however, when established in the experimental field, the presence of phytophagous was notable. The most worrying was *A. spiraecola*, a vector of the citrus tristeza virus; it can colonize different cultivated species. *A. craccivora*, on the other hand, is also a polyphagous species, although it prefers species of the Fabaceae family. It is also an important vector of numerous economically important viruses [36].

Plants such as *Buddleja*, *Baccharis*, and *Senecio* had the highest records of NEs, but also of the aphid species mentioned; however, the fact that they host aphids does not mean that they should be discarded. More studies should be performed to answer questions such as the following: Is the plant a reservoir for the viruses transmitted by the aphid? Or does it only feed on it? Furthermore, since several of the viruses transmitted by aphids are non-persistent [36], the plant could serve as a barrier to clean the aphid’s stylet and thus make it less virulent in crops.

If, in addition to attracting NEs, plants are a source of alternative hosts for parasitoids and predators, the pros and cons of keeping them close to agroecosystems should be assessed. It has been documented that some specialized phytophagous insects of *Asclepias curassavica* (Apocynaceae) function as alternative hosts for NEs, thus enhancing biological control [19]. However, greater efforts are needed to identify phytophagous species associated with nectariferous plants.

The predominant agriculture in Mexico is very particular; for example, there is an ancient agriculture with a broad knowledge of the environment that is practiced by traditional farmers. In various regions of the country, various wild plants are recognized and used either for food or medicine, but there is also knowledge that some wild plants have benefits for their crops. In this way, it can still be observed that vegetation is preserved near or within their crops. In the study region, mesquite trees, *Prosopis* sp.; guamuchil, *Pithecellobium dulce*; and shrubs such as *Senecio* and *Baccharis*, among others, are preserved. Additionally, Mexican agriculture comprises a large tradition of small holder, which allows for a greater number of boundaries between plots. This type of agriculture facilitates the implementation of conservation programs for beneficial insects, as has been suggested for other countries [20].

The list of plants includes trees, shrubs, and annuals, so that, depending on the availability of space and the farmer’s interest in the type of crop, they can select the most suitable plants. Thus, a tree, in addition to preserving NEs and pollinators, can be useful as a living fence post for firewood, windbreak, or shade. A recommendation made by some researchers is to favor shrubs and trees instead of annual herbs; the reasons are that annual plants must be renewed each year [14].

Having plants that flower at different times of the year allows the selection of the appropriate species to guarantee flowering throughout the year and the permanence of NEs near or within agroecosystems. The plants selected here fulfill this purpose. NEs, and particularly the adults of parasitic Hymenoptera, necessarily require nectar and pollen as food to complete their development [2]. If these resources are absent from agroecosystems, NEs populations and the benefits derived from them will be reduced. According to ecological theory [37], after establishing a monoculture, the phytophagous organisms that feed on it (i.e., pests) are the first to arrive; later, the NEs come. If we add alternative foods to natural enemies, we could anticipate their presence before the arrival of pests, with important and predictable benefits for agroecosystems, as has been demonstrated in other studies [12,19,38].

## Figures and Tables

**Table 1:** List of flowering plants from the northwest of Michoacan on which natural enemies were registered during sampling.

Family	Species	Common Name	Tg
Asteraceae	*Cosmos bipinnatus*	Garden Cosmos	A
	*C. sulphureus*	Yellow Cosmos	A
	*Simsia amplexicaulis*	Acahualillo	A
	*Tithonia tubaeformis*	Andan, gigantón	A
	*T. rotundifolia*	Mexican Sunflower	A
	*T. diversifolia*	Tree Marigold	P
	*Verbesina sphaerocephala*	Capitaneja	P
	*Stevia serrata*	Sawtooth Candyleaf	A
	*Stevia* sp.	Candyleaf	A
	*Montanoa* sp.	Palo blanco	P
	*Baccharis salicifolia*	Mule Fat	P
	*Senecio salignus*	Willow Ragwort	P
	*Viguiera quinqueradiata*	Vara blanca	P
Phytolaccaceae	*Phytolacca icosandra*	Tropical Pokeweed	P
Apiaceae	*Coriandrum sativum*	Cilantro	A
Fabaceae	*Eysenhardtia platycarpa*	Kidneywood	P
	*E. polystachya*	Mexican Kidneywood	P
	*Prosopis glandulosa*	Honey Mesquite	P
Sapindaceae	*Thouinia villosa*	Escobetilla	P
	*Serjania racemosa*	Hierba del golpe	P
Brassicaceae	*Lobularia maritima*	Sweet Alyssum	P
	*Rapistrum rugosum*	Bastard Cabbage	A
Tiliaceae	*Heliocarpus terebinthinaceus*	Cicuito	P
Lamiaceae	*Salvia* sp.	Sage	A
Scrophulariaceae	*Buddleja sessiliflora*	Rio Grande butterfly-bush	P
	*B. parviflora*	Tepozan	P

Tg = type of growth; A = annual; P = perennial.

**Table 2:** Predatory insects associated with plant inflorescences in Northwestern Michoacan, Mexico. 2020.

Order	Family	Genus/Species
Neuroptera	Chrysopidae	
	Mantispidae	
Coleoptera	Coccinellidae	*Hippodamia convergens*
		*Stethorus* sp.
		*Scymnus* sp.
		
	Melyridae	
	Staphylinidae	
	Meloidae	
Hemiptera	Anthocoridae	
	Phymatidae	
	Miridae Geocoridae	
Diptera	Syrphidae	
Hymenoptera	Vespidae	
	Formicidae	

**Table 3:** Superfamilies and Families of parasitic Hymenoptera associated with inflorescences of plants from the northwest of Michoacan, Mexico.

Superfamily	Family	Species		
		Number of Individuals	Relative Percentage	Status
Chalcidoidea	Eulophidae	410	74.14	C
	Pteromalidae	12	2.16	C
	Encyrtidae	12	2.16	C
	Mymaridae	10	1.8	C
	Eurytomidae	6	1.08	C
	Torymidae	4	0.72	C
	Aphelinidae	3	0.54	C
	Eupelmidae	3	0.54	C
	Ormyridae	2	0.36	R
	Perilampidae	1	0.18	C
	Trichogrammatidae	1	0.18	C
	Tanaostigmatidae	1	0.18	R
	Eucharitidae	1	0.18	R
Platygastroidea	Platygastridae	47	8.49	C
	Scelionidae	12	2.16	C
Chrysidoidea	Bethylidae	10	1.8	R
Cynipoidea	Cynipidae	9	1.62	C
	Figitidae	1	0.18	R
Proctotrupoidea	Proctotrupidae	3	0.54	C
Ichneumonoidea	Ichneumonidae	2	0.36	C
	Braconidae	1	0.18	C
Ceraphronoidea	Ceraphronidae	1	0.18	C
Apoidea	Sphecidae	1	0.18	C

C = common; R = rare [22].

**Table 4:** Sexual and asexual reproduction of nectariferous species with the greatest potential for the conservation of natural enemies in the northwest of Michoacán. Jiquilpan, Mich., 2020.

Plant Species	Reproduction	
	Sexual (%, n = 100)	Asexual (%, n = 10)
*T. tubaeformis*	8.3	-
*S. serrata*	45.8	-
*Montanoa*	20.8	-
*Baccharis*	Ne	100
*S. salignus*	66.6	-
*V. quinqueradiata*	16.6	-
*P. icosandra*	16.6	-
*E. polystachya*	20.8	-
*T. villosa*	0	-
*S. racemosa*	8.3	-
*R. rugosum*	100	-
*Salvia* sp.	25	-
*B. parviflora*	41.66	-
*Bursera bipinnata*	Ne	100
*B. fagaroides*	Ne	20

Ne = not evaluated.

**Table 5:** Multi-criteria selection of nectariferous plants from the northwest of Michoacán for the conservation of natural enemies.

Species	Parasitoid		Predator	Flowering (Months)	PR	Mult	Rating
	Mean *	Family	Mean *				
*P. icosandra*	0.03	6	0.22	10	0	1	17.2
*S. racemosa*	0.26	10	0.031	5	0	1	16.3
*S. salignus*	0.47	13	0.048	2	−1	1	15.5
*B. salicifolia*	0.23	9	0.14	6	−1	1	15.4
*T. tubaeformis*	2.29	3	2.61	8	−2	1	14.9
*R. rugosum*	0.25	3	0.0	8	0	1	12.2
*V. quin.*	0.66	8	0.23	2	0	1	11.9
*B. parviflora*	1.05	5	0.15	4	−1	1	10.2
*S. serrata*	1.39	6	0.05	1	0	1	9.4
*T. villosa*	0.36	7	0.036	1	0	0	8.4
*Montanoa* sp.	0.18	5	0.033	2	0	1	8.2
*E. polystachya*	0.11	2	0.028	4	0	1	7.1
*P. glandulosa*	0.25	3	0.02	2	0	1	6.3
*V. sphaero.*	0.025	2	0.062	8	−4	-	6.1
*Heliocarpus*	0.1	3	0.072	2	0	-	5.2
*T. rotundi.*	0.07	3	0.07	2	0	-	5.1
*Stevia* sp.	0.07	3	0.0	1	0	-	4.1
*S. amplexicaulis*	0.0	0.0	0.35	4	−1	-	3.3
*Salvia* sp.	0.1	1	0.0	2	0	-	3.1
*E. platycar.*	0.033	1	0.067	1	0	-	2.1
*C. sulphur.*	0.0	0.0	0.037	2	0	-	2.0
*C. bipinnat.*	0.0	0.0	0.033	2	0	-	2.0

* Mean number of individuals/10 inflorescences. PR = phytosanitary risk. Mult = multiplication: 1 = multiplication achieved; 0 = failed to multiply.

## Data Availability

The datasets presented in this article are not readily available due to technical and time limitations. In any case, requests to access the datasets should be directed to the author of the article.
